# Graphene Oxide Functionalized Biosensor for Detection of Stress-Related Biomarkers

**DOI:** 10.3390/s22020558

**Published:** 2022-01-12

**Authors:** Erican Santiago, Shailu Shree Poudyal, Sung Y. Shin, Hyeun Joong Yoon

**Affiliations:** 1 Department of Biomedical Engineering, Michigan Technological University, Houghton, MI 49931, USA; ejsantia@mtu.edu; 2 Department of Electrical Engineering and Computer Science, South Dakota State University, Brookings, SD 57006, USA; poudyal_shree@outlook.com (S.S.P.); Sung.shin@sdstate.edu (S.Y.S.)

**Keywords:** cortisol, graphene oxide, biosensor, electrochemical sensor, point of care

## Abstract

A graphene oxide (GO)-based cortisol biosensor was developed to accurately detect cortisol concentrations from sweat samples at point-of-care (POC) sites. A reference electrode, counter electrode, and working electrode make up the biosensor, and the working electrode was functionalized using multiple layers consisting of GO and antibodies, including Protein A, IgG, and anti-Cab. Sweat samples contact the anti-Cab antibodies to transport electrons to the electrode, resulting in an electrochemical current response. The sensor was tested at each additional functionalization layer and at cortisol concentrations between 0.1 and 150 ng/mL to determine how the current response differed. A potentiostat galvanostat device was used to measure and quantify the electrochemical response in the GO-based biosensor. In both tests, the electrochemical responses were reduced in magnitude with the addition of antibody layers and with increased cortisol concentrations. The proposed cortisol biosensor has increased accuracy with each additional functionalization layer, and the proposed device has the capability to accurately measure cortisol concentrations for diagnostic purposes.

## 1. Introduction

Among the many hormones in circulation throughout the body, cortisol (C_21_H_30_O_5_) is one of the most influential hormones affecting the physiological processes that alter the human body’s homeostasis. Cortisol is classified as a steroid hormone that is synthesized from cholesterol in the zona fasciculata of the kidneys’ adrenal complexes, and it is key to the body’s fight-or-flight state when a stressor occurs [1]. Hence, cortisol has a reputation of being the stress hormone [2]. Under ideal homeostasis, cortisol levels will fluctuate in a day-long cycle, peaking in the morning, and the hormone is released from an unexpected change experienced [3]. As such, cortisol can reach concentrations in the body that are too large or small, resulting in unforeseen effects that may indicate that the glucocorticoid feedback inhibition cycle is impaired [4].

In other studies, cortisol has also been associated with several common stress-based diseases and other disorders. A review conducted by Kiesner and Granger attempted to see if cortisol dysfunction correlated with the onset of premenstrual syndrome and premenstrual dysphoric disorder (PMS/PMDD), but further study was warranted to obtain more conclusive findings [5]. Wei et al. found that cortisol levels in hair samples increased in patients with first-episodic depression, which indicates that cortisol may be a biomarker for depression [6]. Furthermore, a study conducted by Ettman et al. found that depression rates tripled since the onset of the COVID-19 pandemic, making it much more relevant now [7]. Yang et al. found that individuals with Autism Spectrum Disorder (ASD) had higher cortisol and serotonin levels while having lower oxytocin [8]. With increased awareness and criteria changes for ASD diagnoses, cortisol has become more relevant in analyzing ASD. In addition, an influx of evidence suggests that cortisol may be a contributing factor to coronary heart disease (CHD) if it is present in large volumes, especially if maternal cortisol is present during pregnancy [9]. Relatively minor symptoms can also originate from higher and lower cortisol levels affecting (and undermining) the immune system, including insomnia, fatigue, and headaches [10]. Thus, it is necessary to accurately measure cortisol levels at any time of the day while reducing the difficulty in finding cortisol levels.

It is vital to have an accurate measurement at the point of care (POC) to ensure the tested patient’s proper diagnosis and prognosis. Current methods of cortisol testing, such as blood, urine, and saliva tests, are used to detect at least the 10% of cortisol present freely in the blood with methods including immunoassays and liquid chromatography-tandem mass spectrometry (LC-MS/MS) [2,11]. However, it can be a relatively time-consuming process, and some methods must be conducted away from home at an expense to the patient. Furthermore, these tests must be completed at specific times because cortisol levels are ideally higher in the morning and lower in the evening [3]. Apart from collecting a blood sample at a particular time of day, medical professionals may need the patient to collect all their urine in a 24-h period. Some saliva samples may need to be collected at multiple periods throughout the day. These tests are prone to inaccuracies due to the design of the interface between cortisol and quantitative scales for analysis, such as Cohen’s perceived stress scale (PSS) [12]. The result is an overall cost of time and convenience to the patient and healthcare system. However, E. Russel et al. found that cortisol levels in bodily sweat are comparable to those in saliva samples, which indicates that sweat and hair samples could accurately reflect the concentration of cortisol in the body [13].

In recent years, additional research in bio-interfaces has increased for biosensor applications for several different material types. Among these materials, graphene has been a material of interest to researchers and government entities for over four decades [14]. Significant resources and funding have been invested, where the British government alone has invested over 20 million GBP in developing graphene products [15]. The fabled material possesses unique mechanical properties, such as a considerably high mechanical strength of 1 TPa, and it has already seen research and applications for solar cells and nanoelectronic devices [16]. Other mechanical properties associated with graphene include its thermo-conductivity and charge carrier-mobility, measured to be 3000 W/mK and 10,000 cm^2^/V∙s, respectively [16]. One specific variation of graphene comes in the form of graphene oxide (GO), a graphene-based material with oxygen functional groups covalently attached. GO has garnered international interest since the mid-2000s due to the material’s mechanical, electrical, and thermal applications, and it, along with reduced graphene oxide (rGO), has been tested for electrochemical sensors [17]. GO is now a commonplace material for self-assembling monolayers (SAMs), and its properties allow for easy functionalization with other chemicals and biomolecules [18]. This is further justified with GO’s high surface area to volume ratio—specifically a surface area of approximately 2630 m^2^/g—and its ability to function well in aqueous environments [19]. The larger surface area makes it possible for more biomolecules to be functionalized more efficiently. GO is also ideal for medical devices if the sample used on the said device is a liquid (i.e., sweat). The use of GO has been associated with increased specificity in what electrodes and other biological molecules are detected in a sensor, and that application has been exploited in several past studies focusing on cortisol detection and analysis.

Several vital proteins and carbohydrates found in the body can be used as a base to analyze a patient’s physiology. These sensors can detect minute variations in concentrations of various hormones, proteins, and chemicals in real-time with high sensitivity and specificity to their respective applications. An electrode can be designed by coating it in GO and adding specific antibodies to the surface, providing the specificity required by a biosensor [16,20]. Glucose sensors have been designed for research using graphene as done by S. Chaiyo et al., where they developed a paper-based biosensor to detect glucose levels in serum samples [21]. S. Cinti et al. developed a biosensor that detects chloride ions (Cl^−^) using screen-printed filter paper with hydrophilic and phobic sites coated in a sulfuric acid solution with Cl^−^ ions [22]. A lactate biosensor from K. Lin et al. used GO nanosheets coated in a dimethyl-sulfoxide (DMSO) and 1-pyrenebutyric acid–N-hydroxysuccinimide ester (PANHS) to detect lactate concentrations in sweat samples [23]. Concerning cortisol, one application explored by S. Tuteja et al. included a Bluetooth-based means to obtain data from an electro-reduced graphene oxide (e-RGO) sensor using an anti-cortisol antibody CORT-2 and a lactate antibody to detect and isolate cortisol in sweat samples [20]. In addition, more cortisol biosensors were designed by M. Sekar et al. using a conductive carbon fiber material to detect cortisol levels in sweat, and their findings indicated that the sensor was sensitive and specific enough to be properly used specifically for near-complete cortisol detection [24]. A GO biosensor using π-stacked rabbit anti-cortisol antibodies and denatured bovine serum albumin (d-BSA) was created by K. Kim et al. to detect cortisol in saliva samples by utilizing the antibodies’ specificity to detect the hormone [10].

The previously mentioned GO-biosensors maintained their capacities to detect cortisol in serum, sweat, and saliva samples to inform users of potential physiological changes and abnormalities affecting them. To provide a convenient, non-invasive, and swift cortisol test for diagnostic and personalized care applications, research in a sweat-based cortisol biosensor was conducted. With the varying cortisol concentrations throughout the body in mind, this research intended to determine whether a GO interface’s electrochemical responses were affected by those concentrations. These differences in the electrochemical responses based on cortisol concentration may be a determining factor in developing a user-friendly cortisol biosensor for applications at the POC.

## 2. Materials and Methods

### 2.1. Device Principles

**Figure 1:**
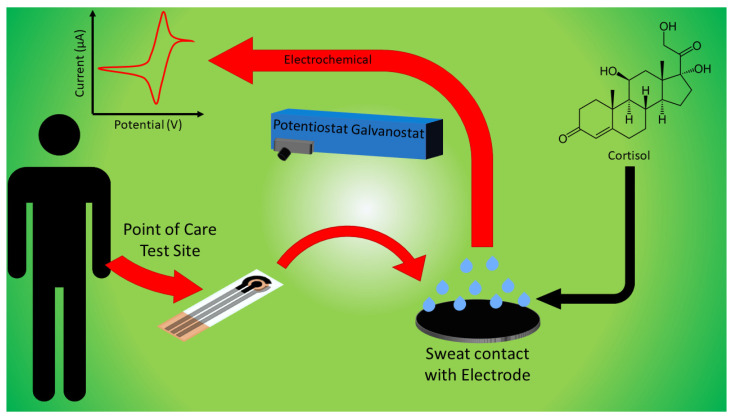
A conceptual graphical abstract of the functionality of the graphene oxide-based biosensor in detecting sweat cortisol for diagnostic purposes.

The GO-based cortisol biosensor is a chemical-sensing device consisting of three electrodes to determine cortisol concentration in the body based on sweat samples. The principal workings of the biosensor are shown below in Figure 1. The device would include a transducer to convert a change in the biological response to an electric signal, including biological elements to detect these changes. In this case, the biosensor is designed to include antibodies. The GO-based sensor also consists of an electrode system using a 3 mm carbon-coated working electrode (WE) and a reference and counter electrode (RE and CE, respectively), which are coated in silver/silver chloride (Ag/AgCl). These Ag/AgCl electrodes were used as the device’s electrochemical transducer by converting physiological changes from sweat samples to a current. The current is also proportional to the cortisol concentration in each sweat sample, indicating that the current will be larger in magnitude as cortisol concentrations are increasing. The sensor is also paper-based to eliminate any use of an external power source but instead relies on capillary action and filtering to achieve similar results.

That said, using GO in this biosensor has a number of advantages and disadvantages to consider. As previously mentioned, GO has a large surface area and is a fabulous conductor of electricity. However, the latter also comes with the drawback of its lack of a band gap—the inability to “switch off” the GO once it begins conducting electricity—due to graphene’s two-dimensional honeycomb lattice structure [25]. Another point of concern for the proposed device is graphene’s susceptibility to oxidative reactions. Although it is oxidized into GO, repeated usage of the biosensor could leave the WE exposed to more oxidative reactions. There are also concerns regarding the possible toxicity of graphene, as there is the possible chance of complications, ranging from irritation and allergic reactions to interactions with dermal proteins [26]. To overcome these obstacles, the sensor was paper-based and intended to be used once to minimize any possible toxic exposure to the skin and oxidizing reactions from the sweat or other biomolecules or chemicals on the skin. It should be noted that the GO on the WE was not directly in contact with the skin but rather the antibodies that were functionalized onto it were.

### 2.2. Biosensor Fabrication

**Figure 2:**
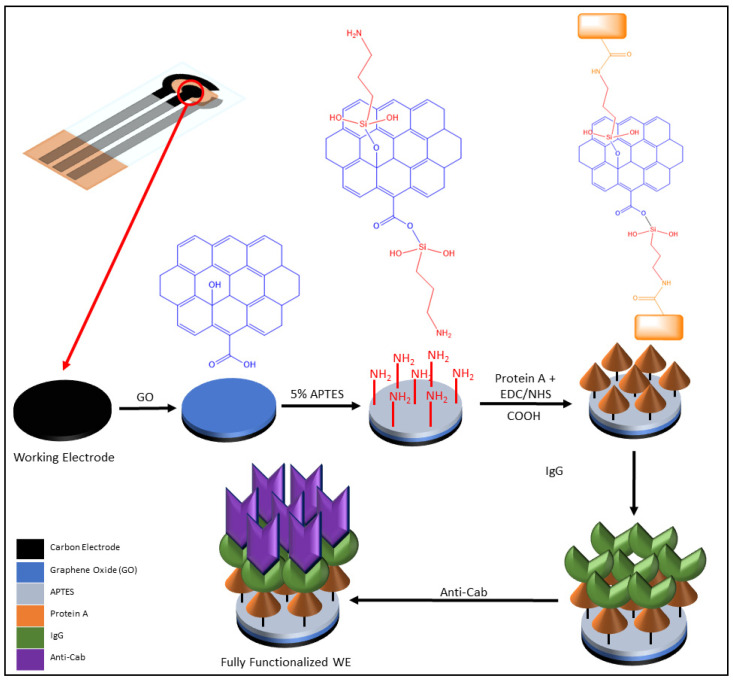
The step-by-step functionalization process for the biosensor’s working electrode to detect cortisol, including the covalent bonding of APTES to graphene oxide and of the C-terminus of Protein A to the free amine group in APTES. Chemical structures drawn using ChemDraw software.

The design of the GO-based cortisol biosensor is highly reliant on the use of self-assembled monolayers (SAMs), specifically on the WE. Past studies have shown that SAMs behave as a novel substrate for any biomolecules captured in the biosensor (i.e., enzymes, antibodies), and that sensor achieves biomimicry of the microenvironment of a cell’s phospholipid bilayer [27,28]. The electrodes in the GO-based sensor are coated in several different layers to fully functionalize the device for cortisol detection (Figure 2). The sensor’s WE was covered with a synthesized microfluidic block to keep any chemical flow through it precise. This microfluidic chamber was prepared in advance of the electrode fabrication and functionalization using polydimethylsiloxane (PDMS) to reduce any redox moieties so an accurate CV measurement could be obtained during experimentation. The PDMS base and curing agent were mixed into a 10:1 ratio and dispensed onto a petri dish and moved to a desiccator to remove any bubbles present in the block. After removing all the bubbles, the chamber was cured in a hot plate for 15–20 min at 65 °C. The resulting block was a sticky PDMS mold that was ready to be cut for the electrodes fabricated. A 3 mm punch was made from the block to cover the WE for the biosensor, and a 6 mm punch was made to protect the WE, RE, and CE electrodes during testing.

The GO solution was prepared by weighing and dispensing 10 mg of GO powder (99 wt% purity, 0.7–1.2 nm thick, 300–800 nm lateral dimensions, 35–42% C, 45–55% O, 3–5% H) to a solution comprised of 10 mL dimethylformamide (DMF) and 300 μL tetrabutylammonium (TBA) hydroxide (40%). DMF was used in the GO solution as part of the required solution composition for CV measurements. For temperature maintenance, the solution underwent tip sonication for 30 min in an ice bucket. The GO solution was incubated at room temperature for two days, and the supernatant in the solution was removed and underwent one hour of bath sonication. Individual samples for the solution were centrifuged at 12,000× *g* rpm for 3 min when the supernatant was once again collected. The supernatant obtained was stored at 4 °C before fabricating the sensor and when not in use. Due to the nature of the functionalization process, photosensitivity was neglected. However, it was essential to maintain the correct temperature while preparing the GO solution properly. To functionalize the WE on the biosensor, 10 μL of GO solution was dispensed through the previously mentioned microchamber to increase the electrode’s surface area and antibody interactions. The electrode was then allowed to incubate at room temperature for one hour, immediately followed by vigorous washing with 10 μL of phosphate buffer saline (PBS) solution three times. The device was given an additional treatment in the form of 10 μL of 5% (3-aminopropyl) triethoxysilane (APTES) solution in acetone being dispensed onto the WE to activate amine groups as APTES binds to the GO SAM (see Figure 2). It then underwent the same incubation and PBS washing procedures previously mentioned.

Protein A was chosen for the biosensor as the first antibody of the functionalization process due to its high affinity. Protein A was selected specifically for its affinity to the constant (Fc) portion of several different species of immunoglobulin macromolecules [29]. A 1-ethyl-3-(3-dimethylaminopropyl) carbodiimide/sulfo-N-hydroxysulfosuccinimide (EDC/sulfo-NHS) solution was prepared for the purpose of activating carboxyl groups as part of the antibody activation process. The EDC/sulfo-NHS allowed for the free and activated amine groups on APTES to form covalent bonds with the carboxyl groups on Protein A’s C-terminuses (see Figure 2). This cross-linking solution was prepared by mixing EDC (4 mg/mL) and sulfo-NHS (11 mg/mL) in a 0.1 M 2-(N-morpholino) ethanesulfonic acid (MES) buffer solution. Following the EDC/sulfo-NHS cross-linking solution synthesis, Protein A was activated by diluting 100 μL of the Protein A stock solution (1 mg/mL) in 890 μL of a 0.1 M MES buffer solution. A total of 10 μL of EDC/sulfo-NHS solution was dispensed into the Protein A solution, and the resulting chemical solution was incubated for 15 min to activate the antibodies’ carboxyl groups. A 160 μg/mL IgG working solution was prepared using a 1.99 mg/mL anti-rabbit IgG stock solution by collecting and dispensing exactly 80.402 μL of the latter solution into 919.598 μL 1% filtered BSA solution.

Once Protein A was fully activated, 10 μL of the Protein A with EDC/sulfo-NHS solution was dispensed onto the WE to bind the Fc binding site on Protein A with the IgG antibody. The Protein A and IgG antibodies were set to incubate at room temperature for an hour, followed by washing the WE with PBS as previously mentioned. A total of 10 μL of anti-Cab antibodies were dispensed onto the WE, incubated for one hour at room temperature, and similarly washed with PBS. The final layer of the WE was prepared using varying cortisol concentrations in PBS solution, including 0.1, 1, 10, 50, and 150 ng/mL, and it was set to incubate at room temperature for 30 min. These varying concentrations were prepared in a Tween-20 and PBS solution. The same PBS washing technique was performed on the WE following incubation. A solution of potassium ferricyanide (K_3_[Fe(CN)_6_]) in PBS was prepared as an electrolyte solution for experimentation. The electrodes were coated with the redox moiety solution to hinder electron transfer to the electrode while measurements were taken.

### 2.3. Experimental Procedures

The cortisol sensors underwent Raman spectroscopy measurements to determine the effectiveness of binding the antibodies onto the screen-printed WE. Three variations of the biosensors were used for the Raman measurements: the sensor with only GO, the sensor with antibodies and GO, and the sensor with only antibodies. A Jobin Yvon Raman LabRAM HR800 System (confocal microscope, 300–1800 g/mm gratings, full area CCD detector, SWIFT-Scan, Horiba, Kyoto, Japan) was used to collect the measurements. All three measurements were done using a 532 nm green laser focused on a point on the electrode. Furthermore, the grating and filter settings were changed, and a 100× lens was used to focus the laser onto the sensors.

Each cortisol sensor underwent cyclic voltammetry (CV) to determine the biosensor’s most optimal functionalization and design. Four electrodes were prepared for CV measurements: GO + APTES, GO + APTES + Protein A, GO + APTES + Protein A + IgG, and GO + APTES + Protein A + IgG + anti-Cab. All CV measurements on the four sensors were performed using a VersaSTAT 4 Potentiostat Galvanostat (2 A maximum current, 2 μs time base, 10 μHz–1 MHz frequency range, AMETEK, Inc., Berwyn, PA, USA) with a potential range of ±0.6 V and a scan rate of 50 mV/s to obtain any well-defined oxidation-reduction peaks. As a means of control, a cortisol biosensor without GO and a biosensor with GO added were both tested for CV measurements with the VersaSTAT 4 Potentiostat Galvanostat. Both sensors were functionalized with the same antibodies regardless of whether they included GO as an initial layer to the WE. They were tested with cortisol samples with a 0.1 ng/mL concentration. The potential range for this test was also set to ±0.6 V to demonstrate the viability of GO in the POC biosensor. To determine the effect of cortisol concentrations on the GO-based biosensor, CV measurements were conducted on the fully functionalized biosensor once again with the VersaSTAT 4 Potentiostat Galvanostat device with a ±0.6 V potential range for the experiment. As previously mentioned, five sensors were tested based on their cortisol concentrations: 0.1, 1, 10, 50, and 150 ng/mL on the WE, and the redox moiety solution was used as the electrolyte solution for all five variations.

Micrographs of the surface of the working electrode at various stages of functionalization were taken with a Hitachi S-4300N scanning electron microscope system. Specifically, two samples were imaged: the working electrode with only GO and the working electrode fully functionalized with all antibodies used. Each sample underwent sputter coating with gold via a CRC-sputtering system to properly image the electrode’s surface. During imaging, a 10 kV voltage was used.

## 3. Results and Discussion

### 3.1. Raman Spectroscopy Measurements

**Figure 3:**
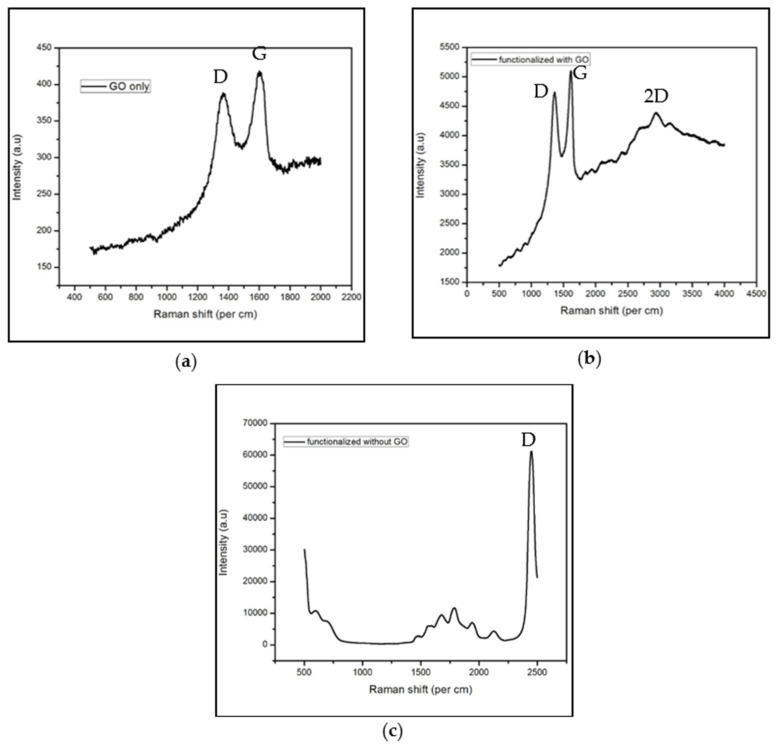
Raman spectrometry measurements on the cortisol biosensors functionalized with (**a**) only graphene oxide, (**b**) both graphene oxide and antibodies, and (**c**) only antibodies.

The Raman spectra for the three sensor variations tested were measured with the LabRAM HR800 system, and notable peaks were detected in all three sensors. Figure 3 features the three individual Raman spectra plotted with respect to wavelength. Specifically, Figure 3a reveals that if the sensor had only GO on its WE, it would have Raman peaks for the D band of GO at 1350 cm^−1^ and the G band at 1600 cm^−1^. The functionalized sensor with GO and antibodies had the largest Raman peaks at 1350 and 1600 cm^−1^ for the GO bands as seen in Figure 3b, and there were additional peaks beyond 1600 cm^−1^ for the antibodies’ individual Raman peaks. Not surprisingly, the sensor without GO and functionalized with antibodies had none of the expected Raman peaks for GO (i.e., no G band). Instead, there were random Raman peaks for each of the antibodies used for the sensors as seen in Figure 3c. The Raman spectroscopy measurements revealed more Raman peaks in the cortisol biosensor with antibodies than those without GO on the WE. In particular, the Raman spectra in Figure 3b is identical to that presented in Khan et al., where the D and G peaks were experienced with approximately the same Raman shifts of 1380 and 1602 cm^−1^, respectively [30]. As there were several peaks with the sensors functionalized with and without GO, the Raman spectra for all three sensors indicated that the anti-Cab antibody had successfully bonded with the carbon WE and other antibodies. This suggests that anti-Cab antibodies appear to be mostly independent of GO when covalent bonding to IgG is concerned.

### 3.2. SAMs Optimization Cyclic Voltammetry Measurements

**Figure 4:**
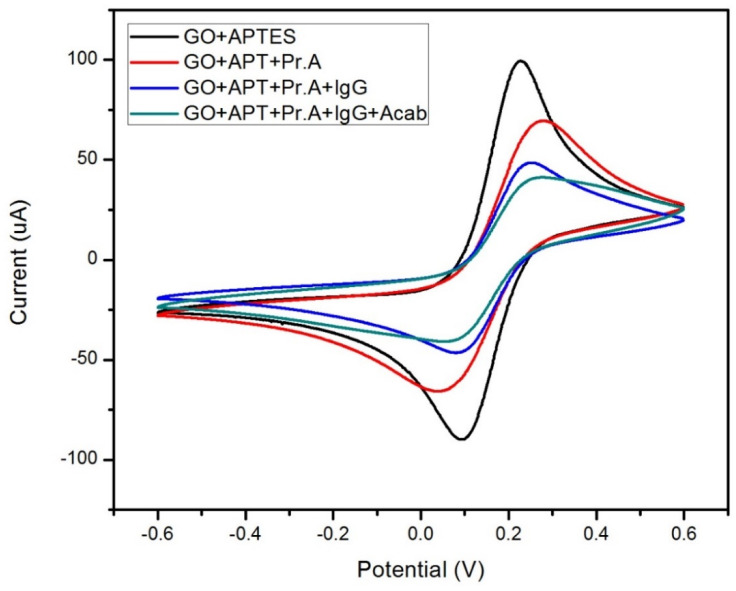
Electrochemical current responses from cyclic voltammetry testing based on additional functionalization layers and groups to the biosensor’s working electrode. A potential range of ±0.6 V was used with K_4_FeCN_6_ in PBS as the redox moiety at a scan rate of 50 mV/s.

As previously stated, CV measurements were conducted with the redox moieties (K_3_[Fe(CN)_6_] in PBS solution) to detect oxidation and reduction from a potential range of ±0.6 V. Figure 4 shows well-defined peaks for oxidation and reduction in all variations of the biosensor. The aforementioned data revealed that each additional layer added to the sensor’s WE during the functionalization process decreased the magnitude of the electrochemical current response in the sensor. This is a considerable improvement over other biosensors, such as the cortisol and lactate biosensor in Tuteja et al., where the increased current response did not have a consistent increase with each additional functionalization layer [20]. That flaw would have made their device more susceptible to data that can be misinterpreted or with a false positive in a clinical setting. Such flaws have been shown in Figure 4 to have been overcome as each CV curve had a consistent decrease in magnitude and were distinguishable from each other.

The sensor’s magnitude decreases indicated that electron transport from K_3_[Fe(CN)_6_] to the electrode was halted after the WE was functionalized with the SAM of the GO material with APTES. The prevention of electron transportation would have been likely caused by the electrostatic interactions between the WE and the positively charged GO-APTES layer on the WE. By dispensing carboxyl-activated Protein A, the amine groups located within the chemical structure of the 5% APTES layer binds with the protein with the assistance of the EDC/sulfo-NHS used prior to activating the carboxyl groups. These chemical bonds are suggested to be the cause of the magnitude decrease for the electrochemical current responses in the sensor. The current magnitude was further decreased by adding the IgG antibodies to Protein A. The protein’s high binding affinity caused the antibodies’ Fc regions due to its charge. The final step in functionalizing the biosensor also lowered the current magnitude with the anti-Cab antibodies reducing electron transport between the SAMs and the WE. These results indicate that each functionalization step used for the device consistently reduces the electrochemical current responses by reducing the transport of electrons between the SAMs.

### 3.3. Demonstration of GO Effects on the Biosensor

**Figure 5:**
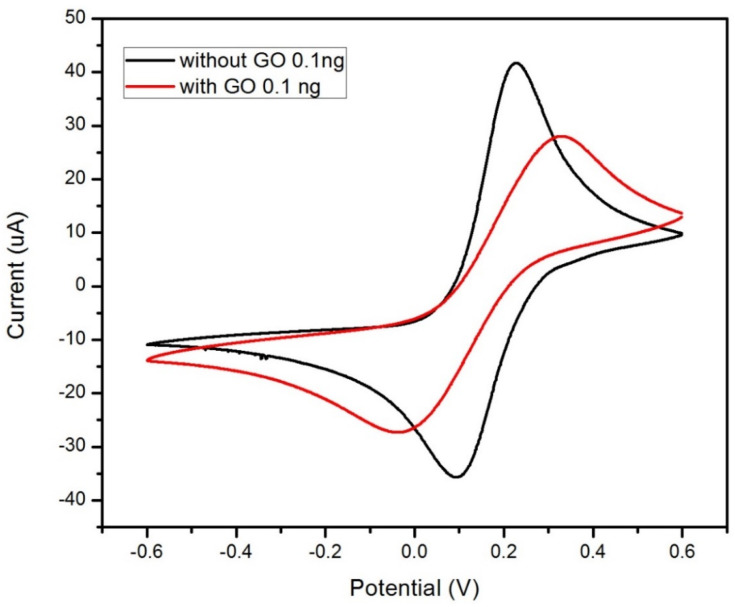
Electrochemical current responses from cyclic voltammetry testing on the cortisol biosensor made with and without 0.1 ng graphene oxide on the working electrode. A potential range of ±0.6 V was used with K_4_FeCN_6_ in PBS as the redox moiety at a scan rate of 50 mV/s.

**Figure 6:**
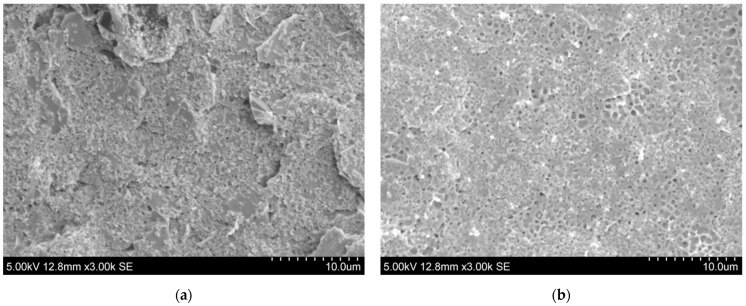
SEM images of the working electrode of the biosensor with (**a**) only graphene oxide and (**b**) full antibody functionalization. Notice the changes in the electrode’s morphology after full functionalization.

CV measurements were performed on two cortisol biosensors to determine the ideal functionality of GO on the WE. Figure 5 displays both current responses generated from the sensors during the test, and the control biosensor without any GO functionalization had a larger current magnitude than that of the GO-based biosensor. The test demonstrated that GO vastly improves the functionality of the cortisol biosensor through the material’s advantageous properties—partially its electrical conductivity properties. GO has a roughness to its surface as proven by Allahbakhsh et al., with a mean surface roughness of 0.619 ± 0.019 nm in multilayered sheets [31], and that surface roughness can be advantageous to the functionalization layers adsorbed to the material. Like the previous test, the reduced current magnitude can also be credited to the reduced number of free electrons present. The roughness would provide additional surface area and affinity for the antibodies on the electrode, resulting in the additional attachment of cortisol to the anti-Cab antibodies. This is confirmed by the findings of Donoso et al., where surface roughness had a positive correlation with surface area [32]. SEM images of the biosensor’s WE were also taken (see Figure 6), and there was a distinct surface roughness to the electrode surface with just GO. When antibodies were added as part of the functionalization process, the surface morphology was much smoother, indicating successful and efficient adsorption of the antibodies onto the electrode, thanks to the GO monolayer.

### 3.4. Cortisol Concentration Cyclic Voltammetry Measurements

**Figure 7:**
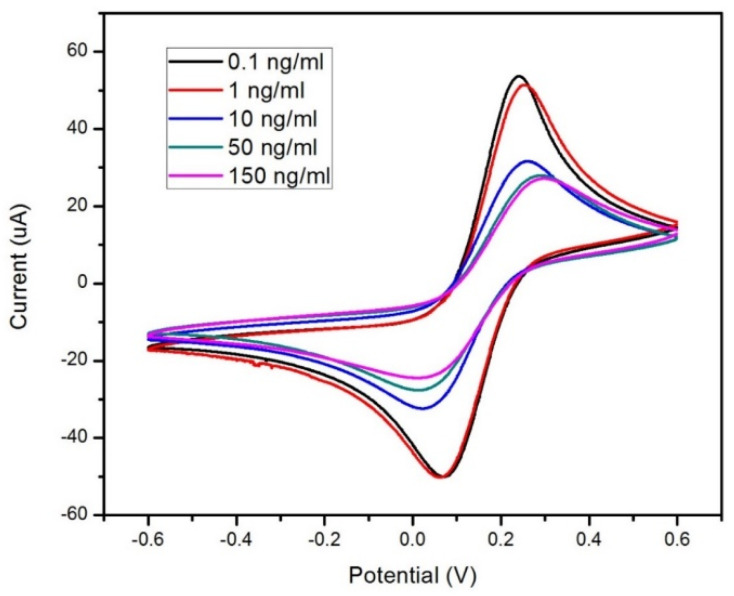
Electrochemical current responses via cyclic voltammetry testing on the biosensor based on cortisol concentrations between 0.1–150 ng/mL. A potential range of ±0.6 V was used with K_4_FeCN_6_ in PBS as the redox moiety at a scan rate of 50 mV/s.

CV measurements were also conducted to analyze the electrochemical response of the GO-based biosensor as a function of varying cortisol concentrations. A total of five different cortisol concentrations were analyzed to compare each concentration’s effect on the sensor’s electrochemical response. Figure 7 depicts these responses with the five concentrations tested on the sensor. It was observed that the magnitude of the current from the electrochemical response decreased in magnitude considerably as the concentration of cortisol increased. Identical to how the current magnitude decreased with each functionalization layer, the decrease was determined to be caused by the addition of the cortisol samples. The anti-Cab antibodies on the GO-based cortisol sensor formed immunocomplexes with the cortisol samples used during experimentation. Thus, cortisol acts as an additional layer to hinder electron transportation between the SAMs in the sensor. Specifically, the higher the cortisol concentrations, the more the hindering effect on electron transport at the electrode–electrolyte interface. This is important, as diminishing electron exchanges would need to be detected by the GO monolayer for the biosensor to work.

## 4. Conclusions

A multi-layer functionalized GO-based biosensor with a carbon WE and Ag/AgCl RE and CE has been proven to determine cortisol concentrations in sweat samples of varying concentrations accurately. The sensor’s current response’s magnitude consistently decreased as additional functionalization layers were added and cortisol concentrations increased. The cortisol concentration in the human body can be distinctively detected at a given POC with the biosensor as an easy-to-use tool for medical and diagnostic purposes. The GO-based biosensor has been designed to address the common concerns and flaws associated with current cortisol detection methods, such as blood and urine analyses. In summation, the proposed medical device permits the distinct electrochemical responses required for an accurate and reliable diagnosis based on a detected concentration of cortisol.

## Data Availability

The data presented in this study are available on request from the corresponding author.
